# Vacuolar ion channels in the liverwort *Marchantia polymorpha*: influence of ion channel inhibitors

**DOI:** 10.1007/s00425-017-2661-4

**Published:** 2017-02-14

**Authors:** Mateusz Koselski, Kazimierz Trebacz, Halina Dziubinska

**Affiliations:** Department of Biophysics, Institute of Biology and Biochemistry, Maria Curie-Skłodowska University, Akademicka 19, 20-033 Lublin, Poland

**Keywords:** Calcium dependence, Chloride channels, Patch-clamp, Pharmacology, SV channels, Vacuole

## Abstract

**Potassium-permeable slow activating vacuolar channels (SV) and chloride-permeable channels in the vacuole of the liverwort** ***Marchantia polymorpha*** **were characterized in respect to calcium dependence, selectivity, and pharmacology.**

The patch-clamp method was used in the study of ion channel activity in the vacuoles from the liverwort *Marchantia polymorpha*. The whole-vacuole recordings allowed simultaneous observation of two types of currents—predominant slow activated currents recorded at positive voltages and fast activated currents recorded at negative voltages. Single-channel recordings carried out in the gradient of KCl indicated that slow activated currents were carried by potassium-permeable slowly activating vacuolar channels (SV) and fast activated currents—by chloride-permeable channels. Both types of the channels were dependent in an opposite way on calcium, since elimination of this ion from the cytoplasmic side caused inhibition of SV channels, but the open probability of chloride-permeable channels even increased. The dependence of the activity of both channels on different types of ion channel inhibitors was studied. SV channels exhibited different sensitivity to potassium channel inhibitors. These channels were insensitive to 3 mM Ba^2+^, but were blocked by 3 mM tetraethyl ammonium (TEA). Moreover, the activity of the channels was modified in a different way by calcium channel inhibitors. 200 µM Gd^3+^ was an effective blocker, but 50 µM ruthenium red evoked bursts of the channel activity resulting in an increase in the open probability. Different effectiveness of anion channel inhibitors was observed in chloride-permeable channels. After the application of 100 µM Zn^2+^, a decrease in the open probability was recorded but the channels were still active. 50 µM DIDS was more effective, as it completely blocked the channels.

## Introduction

Bryophytes were among the first plants that colonized lands. These pioneer plants had to be evolutionally equipped to cope with turbulences in accessibility of water and nutrients. Central vacuoles playing a role of an alternative water environment and possessing appropriate ion channels seem to fulfil an important role in the process of adaptation to the land conditions. The ubiquitous liverwort *Marchantia polymorpha* was the object of our study. This plant was selected by geneticists as a model liverwort and its genome is continuously published. This will soon open the possibility of combining in silico analysis with ion channel behaviour. The only other liverwort whose ion channels in the vacuole have been studied is *Conocephalum conicum* closely related to *M. polymorpha*. In *Conocephalum*, like in most plants tested so far, the Slowly activating Vacuolar channel SV is the most abundant ion channel (Trębacz and Schönknecht 2000; Trębacz et al. 2007). SV channels in *C. conicum* exhibit typical slow kinetics of activation. They are permeable to both mono- and divalent cations. SV channels strongly rectify, allowing cation transport from the cytosol to the vacuolar lumen but not in an opposite direction. What distinguishes SV channels in *C. conicum* from these in vascular plants, is the higher threshold of the cytoplasmic Ca^2+^ concentration [Ca^2+^]_cyt_ necessary for their activation (over 100 µM) and the relatively low impact of luminal calcium [Ca^2+^]_v_ on their open probability (Trębacz et al. 2007). In vascular plants, SV channels are regulated by a plethora of factors including Mg^2+^, Zn^2+^, pH, polyamines, terpenes, choline, dithiothreitol, glutathione, and heavy metals (reviewed by Pottosin and Schönknecht 2007; Hedrich and Marten 2011). A pharmacological approach revealed susceptibility of SV currents to different inhibitors of cation channels from animal cells including tetraethyl ammonium (TEA), amino-acridine, (+)-tubocurarine, quinacrine, and quinidine (Weiser and Bentrup 1993). SV currents were also blocked by ruthenium red, an inhibitor of Ca^2+^ release channels in animal endomembranes (Pottosin et al. 1999). Modulation of the channels, i.e. long-lasting changes in their activity, is induced by phosphorylation/dephosphorylation (Allen et al. 1995; Bethke and Jones 1997), calmodulin (Bethke and Jones 1994), and 14-3-3 proteins (van den Wijngaard et al. 2001).

The discovery that the two-pore channel 1 (TPC1) gene encodes the SV channel protein in *Arabidopsis thaliana* (Peiter et al. 2005) was a milestone that opened examination of the SV/TPC1 channel structure and structure/function relations. Recently, a crystal structure of the channel from *A. thaliana* was published (Guo et al. 2016). The features of SV/TPC1 channels established by electrophysiological experiments are reflected in the structure of the protein (Schulze et al. 2011; Jaslan et al. 2016).

Despite the massive progress in deciphering the structure of the SV/TPC1 channel, its physiological role is still a matter of debate. It is postulated that the channel plays a role of a “safety valve”, which in steady state conditions remains closed. A number of “security” systems in the SV/TPC1 channel serve its opening only in drastic conditions, such as those evoking action potentials (AP). APs in a liverwort closely related to *M. polymorpha*, i.e. *C. conicum*, are generated in response to different kinds of stimuli, including light (Trebacz and Zawadzki 1985), electrical stimulation (Dziubinska et al. 1983), cold (Krol et al. 2003), and menthol (Kupisz et al. 2015). Recently, the role of SV/TPC1 channels in long-distance signalling has been demonstrated (Choi et al. 2014; Kiep et al. 2015). Knockout mutants bearing no functional SV/TPC1 channels were unable to generate Ca^2+^ waves, which usually accompany spreading electrical signals evoked by wounding or herbivorous larvae (Choi et al. 2014; Kiep et al. 2015).

Anion-permeable channels in plant vacuoles are examined much more seldom than cation channels. Most of the data refer to vascular plants. In higher plants, three different types of transporters catalysing anion flux through the vacuolar membrane have been distinguished at the molecular level: aluminium-activated malate transporters (ALMT) (Pantoja and Smith 2002; Hafke et al. 2003; Kovermann et al. 2007), tonoplast dicarboxylate transporters (tDT)—orthologs of animal Na^+^/dicarboxylate exchangers (Emmerlich et al. 2003; Hurth et al. 2005), and chloride channels/exchangers (CLC) (Lv et al. 2009; Isayenkov et al. 2010; von der Fecht-Bartenbach et al. 2010).

Anion-permeable channels in Bryophytes characterized so far have been found in the liverwort *C. conicum* (Trębacz et al. 2007) and the moss *Physcomitrella patens* (Koselski et al. 2015). The channels in *C. conicum* are nearly equally permeable to Cl^−^ and NO_3_^−^ and much less selective to malate. They are activated by an excess of Mg^2+^ at a low concentration of cytoplasmic calcium [Ca^2+^]_cyt_ (Trębacz et al. 2007). It was postulated that Mg^2+^ replaces Ca^2+^ in a putative regulation place. The anion-permeable channels in *P. patens* exhibit high NO_3_^−^ selectivity since the permeability ratio of NO_3_^−^ to Cl^−^ (P_NO3_/P_Cl_) amounted to 3.08. The current flux is directed from the cytosol to the vacuole. The current density decreases at pH below 7.0. The channels require [Ca^2+^]_cyt_ higher than 10 µM and [Mg^2+^]_cyt_ above 2 mM for activation (Koselski et al. 2015). In silico research indicated homology between CLC-type proteins in *Arabidopsis* and in *Physcomitrella* (Koselski et al. 2015).

This is the first study concerning biophysical characterization of ion channels in *Marchantia* vacuoles with the application of the patch-clamp technique. Special emphasis was paid to SV and anion channels.

## Materials and methods

### Plant material

Thalli of *M. polymorpha* were collected in the Botanical Garden of Maria Curie-Skłodowska University in Lublin. Gemmae were taken from the gemma-cups of male plants and placed on peat pellets for cultivation. The plants were cultivated in a vegetative chamber at 23 °C, humidity 50–70%, and under a 16:8 h (light:dark) photoperiod with the light intensity of 20–40 μmol m^−2^ s^−1^. Four to five-week-old plants were used for electrophysiological experiments.

### Vacuole isolation

The vacuoles were isolated with the non-enzymatic method described by Trębacz and Schönknecht (2000). Before the experiments, a fragment of a thallus cut from a rhizoid-free area was plasmolysed in a bath medium supplemented with 500 mM sorbitol. After 20–30 min, a fragment of the thallus was cut with a razor blade and transferred to a measuring chamber containing a solution with an osmotic pressure of 500 m Osm kg^−1^ (the value of this parameter in the micropipette was 550 m Osm kg^−1^). In such an osmotic pressure, the deplasmolysis of the cells caused release of the protoplast from the cut-off cell walls. After a few minutes, some of the protoplasts ruptured and release of vacuoles was observed.

### Patch-clamp experiments

The patch-clamp experiments were performed in the whole-vacuole and cytoplasm-out configuration. The micropipettes were made from borosilicate tubes (Kwik-Fil TW150-4; WPI, Sarasota, FL, USA), which were pulled and fire polished by a DMZ-Universal Puller (Zeitz-Instruments, Martinsried, Germany). An Ag/AgCl reference electrode filled with 100 mM KCl was connected with the bath solution by a ceramic porous bridge. A cryoscopic osmometer (Osmomat 030; Gonotec, Berlin, Germany) was used for checking the solution osmolarity. The experiments were performed using an EPC-10 amplifier (Heka Electronik, Lambrecht, Germany) working under the Patchmaster software (Heka Electronik). The recordings were sampled at 10 kHz and filtered with 1 kHz. The solution in the measuring chamber was exchanged before recording by a peristaltic pump (ISM796B; Ismatec, Wertheim, Germany). The results were presented according to the convention proposed by Bertl et al. (1992).

### Analysis of the results

Current density/voltage (J/V) and current/voltage (I/V) characteristics were prepared in SigmaPlot 9.0 (Systat Software Inc.). The amplitude histograms were fitted in GRAMS/AI 8.0 (Spectroscopy Software). The open probability of the channels was calculated from the area under the Gaussian peaks. The reversal potentials (*E*_rev_) were calculated based on the activities of the ions used. Due to the non-linear *I*/*V* characteristics, the unitary conductance was calculated as a *I*/*V* ratio obtained at the specific voltage. The number of repeats (*n*) indicates the number of tested tonoplast patches or whole vacuoles.

## Results

**Fig. 1:**
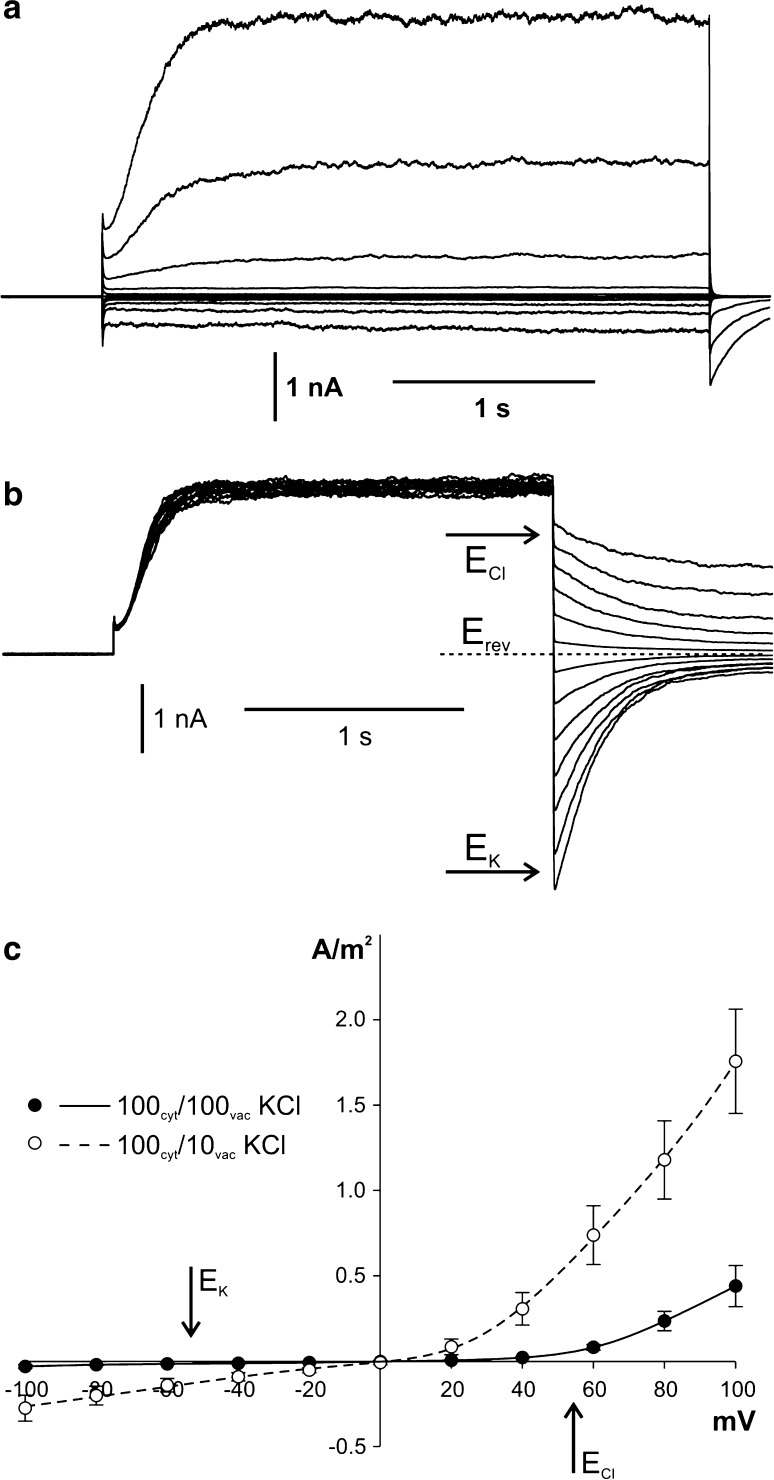
Activity of ion channels recorded in the whole-vacuole configuration. **a** Recordings obtained in the symmetrical concentration of 100 mM KCl (the pipette contained 100 mM KCl, 0.1 mM CaCl_2_, 2 mM MgCl_2_, pH 5.5 buffered by Hepes/TRIS, and the bath—100 mM KCl, 0.1 mM CaCl_2_, 2 mM MgCl_2_, pH 7.2 buffered by Mes/TRIS). Recordings were obtained by application of 0.5 s holding voltage (0 mV), then 3 s test voltages with 20 mV steps from 100 to −100 mV, and 0.3 s pulse (0 mV) after the test voltage. **b** Tail currents recorded in the gradient of KCl obtained after reduction of KCl in the pipette to 10 mM (the pipette contained 10 mM KCl, 0.1 mM CaCl_2_, 2 mM MgCl_2_, pH 5.5 buffered by Hepes/TRIS). Recordings were obtained by application of 0.5 s holding voltage (0 mV), then 2 s voltage which activated SV channels (80 mV), and 1 s test voltages with 10 mV steps from −60 to 60 mV. The reversal potential (*dashed line*) and the equilibrium potential for K^+^ and Cl^−^ (*arrows*) are indicated. **c** *J*/*V* curves obtained in the symmetrical concentration of 100 mM KCl (the same conditions as in **a**, *closed circles* and *solid line*, *n* = 6) and after reduction of the KCl concentration in the pipette to 10 mM (the same conditions as in **b**, *open circles* and *dashed lines*, *n* = 7)

**Fig. 2:**
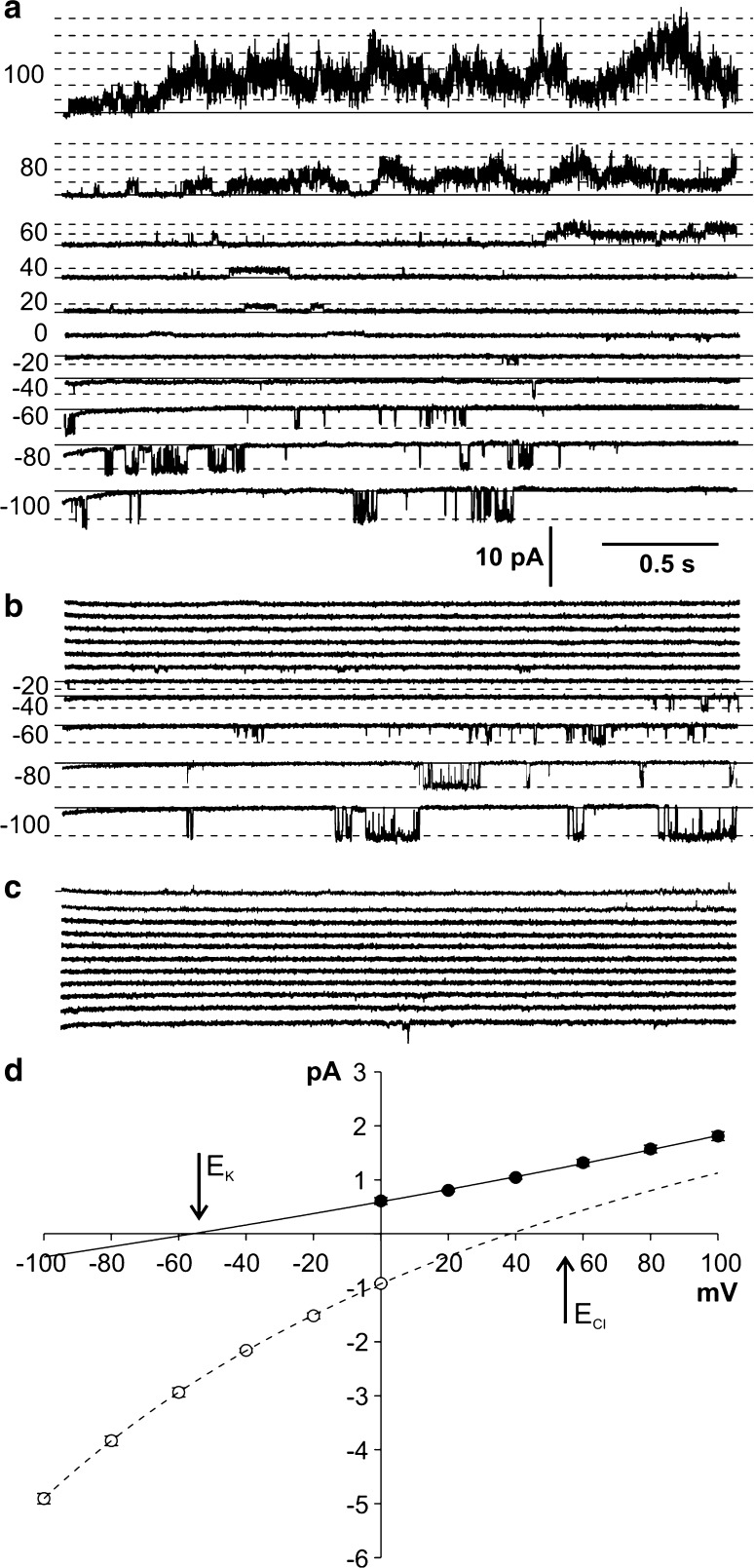
Activity of ion channels recorded in the cytoplasm-out configuration. **a** Recordings obtained in the gradient of KCl (the pipette contained 10 mM KCl, 0.1 mM CaCl_2_, 2 mM MgCl_2_, pH 5.5 buffered by Hepes/TRIS, and the bath—100 mM KCl, 0.1 mM CaCl_2_, 2 mM MgCl_2_, pH 7.2 buffered by Mes/TRIS). *Solid lines* indicate the close state, and *dashed lines*—open states of the channels. The holding voltages are placed on the *left side* of the traces. **b** Recordings obtained at the same patch after replacement of 100 mM KCl in the bath by 100 mM HCl (100 mM HCl, 0.1 mM CaCl_2_, 2 mM MgCl_2_, pH 7.2 buffered by BIS–TRIS Propane). **c** Recordings obtained after replacement of 100 mM HCl in the bath by 100 mM K-gluconate (100 mM K-gluconate, 0.1 mM CaCl_2_, 2 mM MgCl_2_, pH 5.5 buffered by Hepes/TRIS. **d** *I*/*V* curves obtained in the same conditions as in **a**. *Close circles* and the *solid line* denote currents flowing through SV channels (*n* = 10), and *open circles* and the *dashed line* denote currents flowing through chloride channels (*n* = 11)

The whole vacuole measurements carried out in symmetrical (in the bath and in the pipette) 100 mM KCl allowed observation of slowly activated positive currents and fast activated negative currents (Fig. 1a). Slow kinetics of activation was observed especially at voltages higher than 40 mV; it consisted in an increase in the current density recorded during several hundreds of milliseconds after application of the voltage pulse. The density of the fully activated positive currents measured from the marginal part of the recordings (80–95%) obtained at 100 mV amounted to 0.44 ± 0.12 A/m^2^ (*n* = 6) and was considerably higher than the negative currents recorded at −100 mV (0.03 ± 0.01 A/m^2^, *n* = 6). Higher densities of both currents were recorded when the concentration of KCl in the pipette was reduced to 10 mM (Fig. 1c). Application of the KCl gradient did not cause a shift of the reversal potential measured at the steady state currents, which remained close to zero. A similar value of the reversal potential was also obtained from the tail-current analysis (Fig. 1b). The lack of the reversal potential shift recorded in the KCl gradient indicated simultaneous activity of two types of ion channels with different selectivity for K^+^ and Cl^−^ in the tonoplast of *Marchantia*. If both K^+^ and Cl^−^ ions flowed through the tonoplast in the same direction, the whole-vacuole outward currents could be balanced by inward currents. To examine such a possibility, we carried out measurements in the cytoplasm-out configuration, which allowed us to record the activity of single ion channels (Fig. 2). The cytoplasm-out measurements showed that channels recorded at positive and negative voltages had different conductance, which at 100 mV amounted to 18 ± 1 pS (*n* = 8) and at −100 mV to 49 ± 1 pS (*n* = 11). Moreover, at negative voltages, usually one channel was active whilst the number of active channels at positive voltages increased together with the magnitude of the applied voltage (Fig. 2a). The amplitudes of recorded currents allowed drawing two *I*/*V* curves, separately for positive and negative currents, which crossed the abscissa at different voltages indicating simultaneous activity of two types of channels with different selectivity (Fig. 2d). The values of the reversal potential estimated from the *I*/*V* curves amounting −56 and 38 mV were close to the reversal potentials for potassium (*E*_K_ = −55 mV) and chloride (*E*_Cl_ = 55 mV), respectively. Such results indicated that, at positive voltages, the activity of outwardly rectifying K^+^-permeable channels was recorded; in turn, at negative voltages, Cl^−^-permeable channels were active. The potassium and chloride selectivity of the channels was confirmed in experiments that imposed reduction of K^+^ and Cl^−^ fluxes from the bath (cytoplasmic side) to the pipette (vacuolar side) by replacement of K^+^ or Cl^−^ in the bath by ions impermeable to potassium or chloride channels, respectively. Elimination of potassium currents was achieved by replacement of 100 mM KCl by 100 mM HCl (Fig. 2b). The inactivation of positive currents recorded in the absence of K^+^ in the bath did not inhibit the negative currents, whereas the currents were completely blocked after the replacement of 100 HCl by 100 mM K-gluconate (Fig. 2c).

**Fig. 3:**
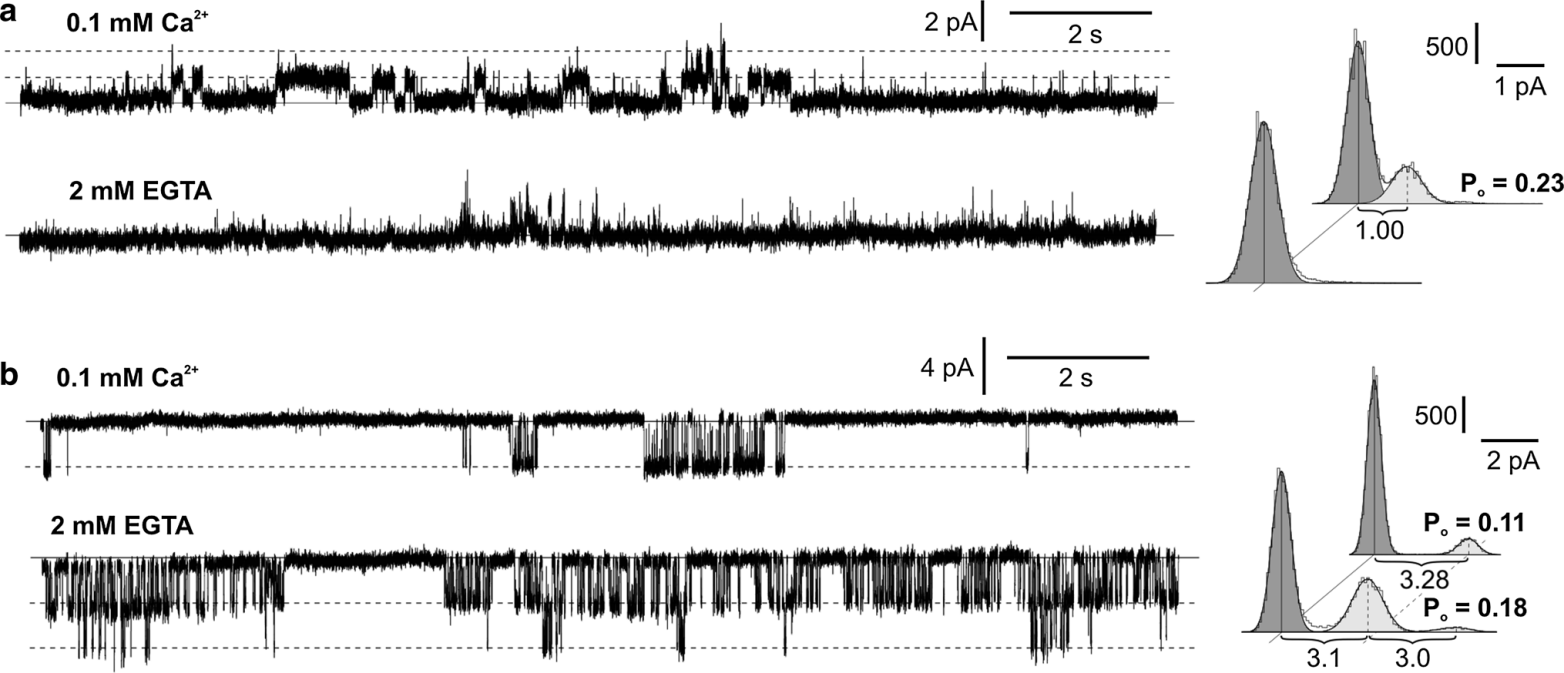
Calcium dependence of SV channels (**a**) and chloride-permeable channels (**b**). The cytoplasm-out recordings were obtained at the same patch at +40 mV (**a**) and −60 mV (**b**). The traces described as 0.1 Ca^2+^ were obtained in the same conditions as in Fig. 2a. The amplitude histograms indicating the current amplitude (*horizontal line*) and the number of sample points (*vertical line*) correspond to the traces obtained in standard conditions (*upper histogram*) and after elimination of cytoplasmic calcium by application of 2 mM EGTA (*lower histogram*).The *diagonal dashed lines* indicate open states and the *solid line*—close states of the channels. The values of the current amplitude and the open probability (Po) were indicated

Many types of vacuolar ion channels are calcium dependent. A majority of vacuolar channels are active in the presence of cytoplasmic calcium and such dependence occurs in SV channels (Allen and Sanders 1996; Pottosin and Schönknecht 2007; Trębacz et al. 2007; Hedrich and Marten 2011; Koselski et al. 2013;), potassium-selective vacuolar channels (VK) (Ward and Schroeder 1994; Allen and Sanders 1996; Allen et al. 1998; Pottosin et al. 2003), fast activated vacuolar channels (FV) (Tikhonova et al. 1997; Allen et al. 1998), one of ALMTs from *A. thaliana* (AtALMT6) (Meyer et al. 2011), chloride channels from *Vicia faba* activated by calcium-dependent protein kinase (CDPK) (Pei et al. 1996), and nitrate-permeable channels from *P. patens* (Koselski et al. 2015). The calcium dependence of the ion channels from *Marchantia* was studied by elimination of 0.1 mM Ca^2+^ from the cytoplasmic side (Fig. 3). The measurements were carried in a cytoplasm-out configuration by application of a long lasting (16 s) voltage pulse, which was close to the reversal potentials obtained previously. The selected values of voltage pulses, +40 and −60 mV, guaranteed avoidance of the activity of chloride and potassium channels, respectively. The results obtained in the absence of cytoplasmic calcium indicated different calcium dependence of potassium- and chloride-permeable channels. The inhibition of potassium-permeable channels recorded in the absence of cytoplasmic calcium (Fig. 3a) allowed us to classify the channels to SV channels, the most popular slow activated and calcium-dependent vacuolar cation-permeable channels. An opposite effect was recorded in the case of fast activated chloride-permeable channels, since elimination of cytoplasmic calcium not only did not inhibit the channels but also evoked an increase in the open probability (Fig. 3b). Such results are further evidence for existence of two different channels in the vacuoles of *Marchantia*, i.e. SV channels and chloride-permeable channels.

**Fig. 4:**
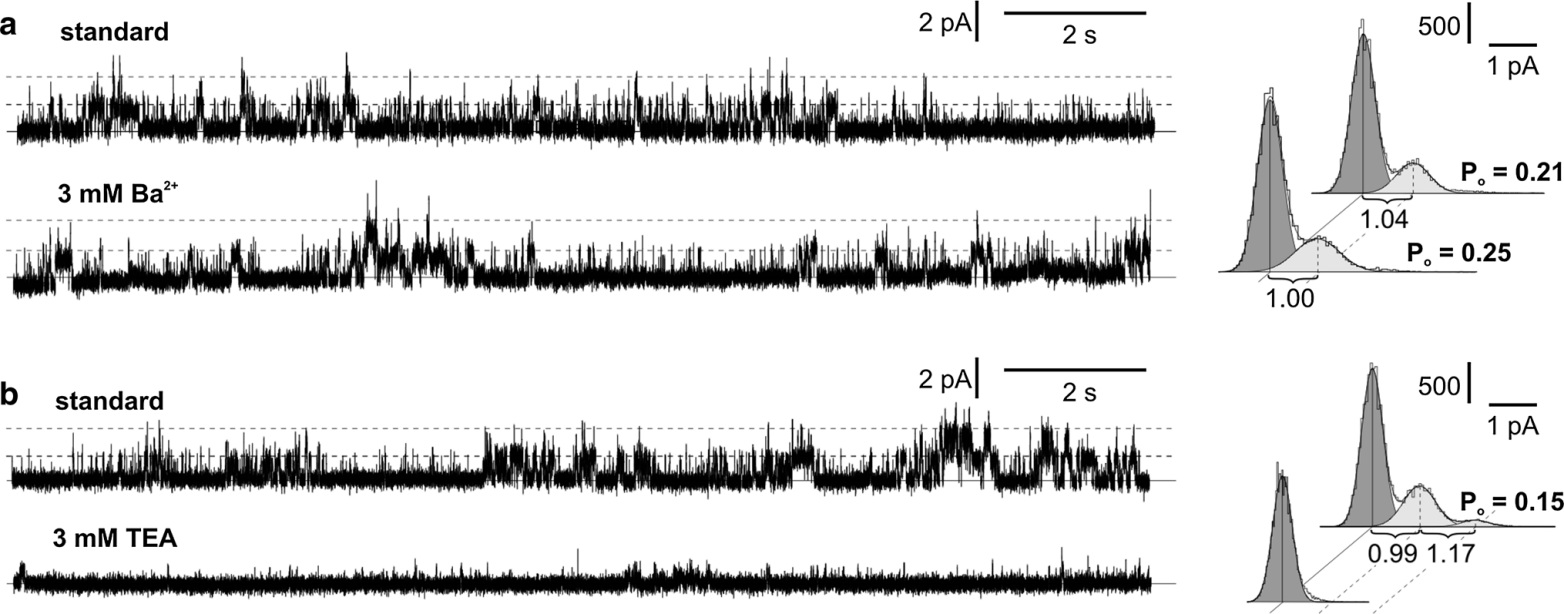
Influence of potassium channel inhibitors, BaCl_2_ (**a**) and TEA (**b**) on the activity of SV channels. The standard conditions were the same as in Fig. 2a. The cytoplasm-out recordings were obtained at the same patch at +40 mV. The amplitude histograms correspond to the traces obtained in standard conditions (*upper histogram*) and after application of the inhibitor to the bath solution (*lower histogram*)

**Fig. 5:**
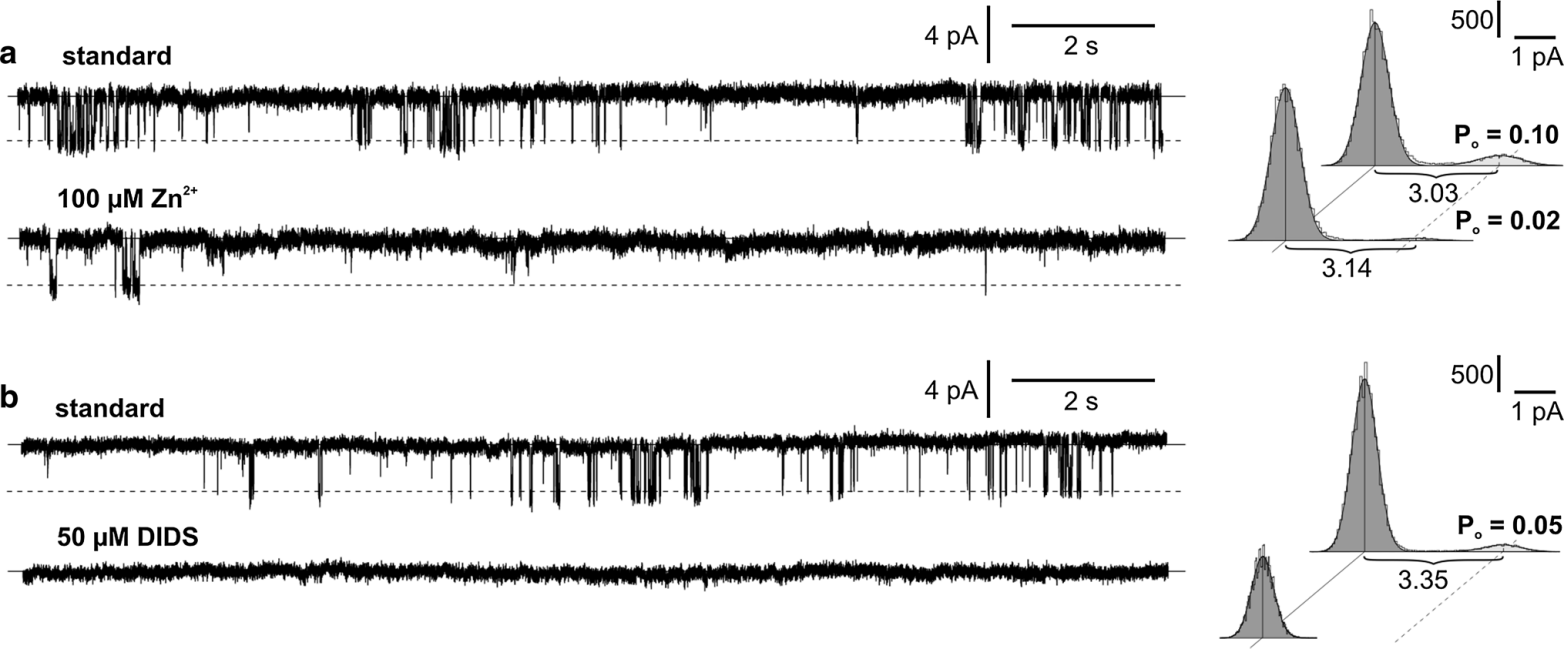
Influence of chloride channel inhibitors, ZnCl_2_ (**a**) and DIDS (**b**) on the activity of chloride channels. The standard conditions were the same as in Fig. 2a. The cytoplasm-out recordings were obtained at the same patch at −60 mV

Ion channels from *Marchantia* were characterized in more detail by using ion channel inhibitors. Different effectiveness in blockage of SV channels was obtained after the application of two inhibitors, BaCl_2_ (3 mM) and TEA (3 mM) (Fig. 4). BaCl_2_ almost unchanged the activity of the SV channels. According to the amplitude histogram, application of BaCl_2_ caused slight changes in the open probability (an increase from 0.21 to 0.25) and conductance (a decrease from 1.04 to 1.00 pA) (Fig. 4a). In turn, total blockage of SV channels was obtained after application of TEA (Fig. 4b). Different effectiveness of two anion channel inhibitors, ZnCl_2_ (100 µM) and 4,4′diisothiocyanatostilbene-2,2′-disulfonic acid (DIDS; 50 µM), was recorded in respect to the chloride channel activity (Fig. 5). Application of ZnCl_2_ did not block the channels but lowered their open probability from 0.10 to 0.02 (Fig. 5a). The effects were observed a few minutes after application of ZnCl_2_. DIDS was more effective and evoked total blockage of the channels immediately after application (Fig. 5b).

**Fig. 6:**
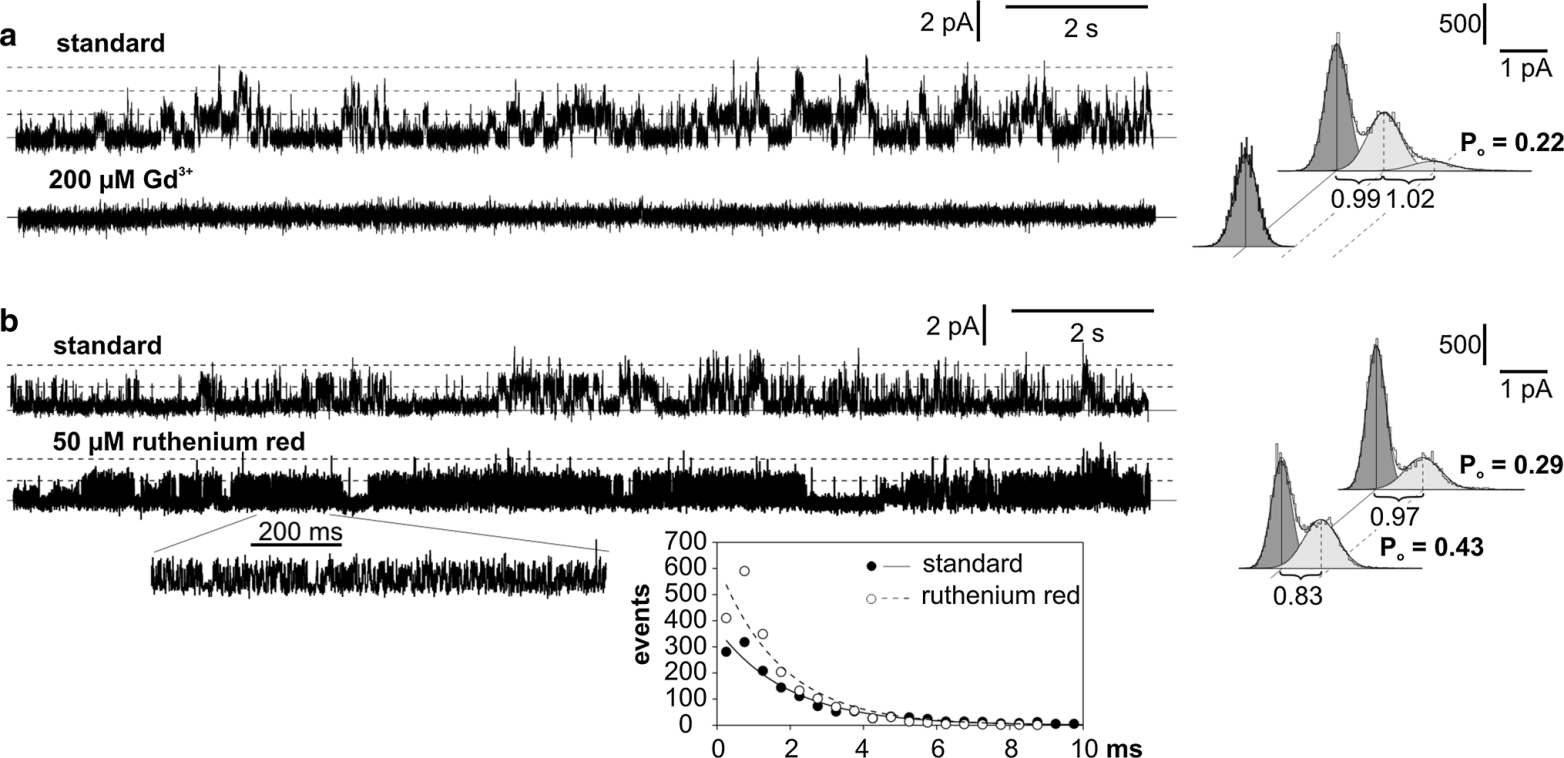
Influence of calcium channel inhibitors, GdCl_3_ (**a**) and ruthenium red (**b**) on the activity of SV channels. The standard conditions were the same as in Fig. 2a. The cytoplasm-out recordings were obtained at the same patch at +40 mV. The event detection analysis placed at the *bottom* of **b** was based on the presented traces

Since SV channels are known to be permeable to mono- and divalent cations including calcium, it was interesting to examine if SV channels from *Marchantia* share common features with calcium channels and are sensitive to calcium channel inhibitors. We decided to study the sensibility of the channels on two calcium channel inhibitors: GdCl_3_ (200 µM) and ruthenium red (50 µM). Complete blockage of SV channels was recorded after application of 200 µM GdCl_3_ (Fig. 6a). In comparison to gadolinium, ruthenium red evoked almost opposite effects to the activity of the SV channels (Fig. 6b). In a majority of the tested patches (5 of 7), this inhibitor evoked bursts of rapid flickering of SV channels between open and close states. This phenomenon was accompanied by an increase in the open probability from 0.29 to 0.43. The event detection analysis indicated that ruthenium red evoked an increase in the short-lasting (up to ca. 5 ms) openings of the channels (see the inset in Fig. 6b). The next effect observed after ruthenium red was a decrease in the conductance of the channels from 24 to 21 pS.

## Discussion

The results presented in this work demonstrate that at least two channel types are active in the tonoplast of the liverwort *M. polymorha*—slow activated potassium-permeable channels and fast activated chloride-permeable channels. The slow activated channels found in *Marchantia* share many features with SV channels—the best known plant vacuolar channels found also in species related to *Marchantia*, i.e. *C. conicum* (Trębacz and Schönknecht 2000; Trębacz et al. 2007) and *P. patens* (Koselski et al. 2013). The same channels in *A. thaliana* are encoded by the AtTPC1 gene, since *tpc1* knockout mutants exhibit lack of SV channel activity (Peiter et al. 2005). In *Marchantia*, similar as in other plants, SV channels are outward rectifiers and allow potassium to pass from the cytoplasm to the vacuole. Moreover, the channels possess another feature that is characteristic for SV channels—dependence on cytoplasmic calcium (Fig. 3a). In comparison to potassium channels, classification of chloride channels is more difficult, because vacuolar anion channels in plant cells are not as well known as SV channels. Vacuolar anion channels were recorded previously in *C. conicum* (Trębacz et al. 2007), but they were activated by a high cytoplasmic concentration of magnesium (50 mM) and a low concentration of Ca^2+^ (not added to the cytoplasmic side). Anion channels from *Conocephalum* were permeable to many anions, including chloride, and carried the ions in the same direction as the channels in *Marchantia*. Both channels differed in conductance, which at −100 mV amounted to 32 pS in *Conocephalum* and 49 ± 1 pS in our study (Fig. 2d). However, such differences could be an effect of different conditions used during the measurements. The anion channels in *Conocephalum* were recorded in an almost symmetrical concentration of 100 mM Cl^−^ (cytoplasmic 100 mM Cl^−^ and vacuolar 104 mM Cl^−^); in our study, an approximately tenfold lower concentration of Cl^−^ was used on the vacuolar side, facilitating Cl^−^ fluxes toward the vacuole. Substantially higher conductance than in *Marchantia* was recorded in the anion-permeable vacuolar channels from *Physcomitrella* (108 pS obtained at −80 mV in symmetrical 208 mM Cl^−^) (Koselski et al. 2015). These channels were permeable to nitrate and chloride with a NO_3_^−^/Cl^−^ permeability ratio of 3.08 and carried the anions in the same direction as the anion channels from *Marchantia* and *Conocephalum*. The channels from *Physcomitrella*, unlike in our study, were recorded after inhibition of SV channels. Such dependence of anion channels and SV channel activity was not observed in *Marchantia*, since both the channels were recorded simultaneously (Fig. 2a). The next difference between the anion channels recorded in *Physcomitrella* and *Marchantia* is calcium dependence. While the anion channels in *Physcomitrella* needed 100 µM cytoplasmic Ca^2+^ for activation, a change of the cytoplasmic calcium concentration from 100 µM to 0 in *Marchantia* evoked an increase in the open probability (Fig. 3b). Besides the vacuolar chloride-permeable channels found in species closely related to *Marchantia*, it is worth mentioning a member of a family of ALMTs–AtALMT9, characterized in the vacuoles of *A. thaliana* (Kovermann et al. 2007; De Angeli et al. 2013b) and *Vitis vinifera* (De Angeli et al. 2013a). This channel was permeable to chloride, and according to patch-clamp recording carried out on *Arabidopsis*, its conductance obtained in a symmetrical concentration of 100 mM Cl^−^ amounted to 32 pS (the same value was obtained in *Conocephalum*). The channel was an inward rectifier and carried chloride in the same direction as each of the anion channels mentioned above, but again its activation was dependent on cytoplasmic malate. In turn, another member of ALMT found in the vacuole of *Arabidopsis*—AtALMT6 was also an inward rectifier, but carried mainly malate and was dependent on cytoplasmic calcium (Meyer et al. 2011). Different ways of activation of plant vacuolar anion channels noted even in closely related species indicate substantial differentiation of these kinds of channels.

The occurrence of potassium- and chloride-permeable channels in *Marchantia* was confirmed in this study first by the values of reversal potentials obtained in the KCl gradient (Fig. 2d) and then by the inhibition of potassium or chloride currents observed after the elimination of K^+^ or Cl^−^ in the bath (Fig. 2b, c). Supplementary results about the pharmacology of the recorded channels were obtained in the presence of inhibitors of calcium, potassium, and chloride channels. The most variable effects were evoked by two calcium channel inhibitors (Gd^3+^ and ruthenium red), which in different ways changed the activity of the SV channels (Fig. 6). Gadolinium was the most effective inhibitor. The same inhibitor was an effective blocker of calcium channels in the endoplasmic reticulum from touch-sensitive tendrils of *Bryonia dioica* (Klusener et al. 1995). These channels tested with the lipid bilayer technique were voltage dependent and were probably involved in calcium-based mechanotransduction, since gadolinium abolished the response to touch. The second calcium channel inhibitor used in our study—ruthenium red, evoked long lasting bursts of the channel activity, during which rapid flickering of SV channels was recorded (Fig. 6b). Such flickering of channels after application of ruthenium red was also recorded in SV channels from *Beta vulgaris* (Pottosin et al. 1999). In these channels, ruthenium red applied on the cytoplasmic side at a concentration of 0.1–1 µM evoked two modes of activity depending on the applied voltage: long-term closures of the channels recorded at voltages lower than ca. 50 mV and blocks of flickering of the channels recorded at more positive voltages. At a higher concentration of ruthenium red (3–5 µM), SV channels from *Beta vulgaris*, in contrast to SV channels from *Marchantia*, were blocked. Apart from SV channels, the ryanodine receptor (RyR), which acts in the animal storage/release organelles as a Ca^2+^ release channel (Fill and Copello 2002), is sensitive to ruthenium red (Xu et al. 1999). However, no RyR homolog has been found in land plants (Schönknecht 2013).

The SV channels in *Marchantia* were blocked by TEA (Fig. 4b), i.e. an inhibitor commonly used for blocking of many types of potassium channels. TEA blocks SV channels from higher plants e.g. *Chenopodium rubrum* (Weiser and Bentrup 1993) and *Beta vulgaris* (Hedrich and Kurkdjian 1988). Weak effectiveness of this inhibitor (reduction of whole-vacuolar SV currents by 18% after application of 10 mM TEA) was recorded in the green alga *Eremosphaera viridis* (Linz and Köhler 1994). On the other hand, a patch-clamp study carried out by Schulzlessdorf and Hedrich (1995) proved TEA-permeability of SV channels from guard cells of *Vicia faba*. Interestingly, the SV channels from *Marchantia* were not blocked by another inhibitor of potassium channels—barium, which at the 10 mM concentration inhibited SV currents from *Beta* with higher efficiency (50–70% of inhibition) than 10 mM TEA (20–50% of inhibition) (Hedrich and Kurkdjian 1988). On the other hand, a patch-clamp study carried out by Pantoja et al. (1992b) indicated that SV channels from *Beta* are permeable to barium. Apart from barium, there are many monovalent (K^+^, Na^+^, Rb^+^, Cs^+^) and divalent (Ca^2+^, Mg^2+^) cations for which low selectivity of SV channels has been documented (White 2000). Probably, low selectivity to cations occurs also in the SV channels from *Marchantia*. This assumption is supported by the differences in *E*_K_ and *E*_rev_ obtained in the K^+^ gradient (Fig. 2d), but in the presence of the symmetrical concentration of Ca^2+^, whose fluxes through SV channels were documented previously in *P. patens* (Koselski et al. 2013), *Vicia faba* (Schulzlessdorf and Hedrich 1995; Ward and Schroeder 1994), and *Beta vulgaris* (Pottosin et al. 2001). Low selectivity and permeability to mono- and divalent cations, if existing in the SV channels from *Marchantia*, can explain the lack of blockage of these channels by barium.

To date, there is insufficient knowledge about the influence of anion channel inhibitors on the activity of anion channels from the vacuole. Two of the known anion channel inhibitors, DIDS and Zn^2+^, were effective in blocking the activity of chloride-permeable channels in *Marchantia* (Fig. 5). The channel activity was also abolished by replacement of Cl^−^ by gluconate, which is impermeable to chloride channels (Fig. 2c). The effectiveness of some anion channel inhibitors, including DIDS, was also confirmed in vacuolar anion channels from *Conocephalum* (Trębacz et al. 2007). Besides blocking the chloride channels from *Marchantia*, Zn^2+^ was also an effective blocker of malate-permeable vacuolar channels from *Beta* (Pantoja et al. 1992a), which was considered as the route for malate movement into the vacuole. Together with the above-mentioned results obtained in *Conocephalum* and *Beta*, our investigation is so far the only study that concerns the pharmacology of plant vacuolar anion channels.

Tables 1 and 2 were prepared to facilitate the comparison of the effects of calcium, potassium, and anion channel inhibitors in *Marchantia* and other plants described earlier.

**Table 1:** Effects of potassium channel inhibitors (Ba^2+^ and TEA) and calcium channel inhibitors (Gd^3+^ and ruthenium red) on the activity of potassium and calcium permeable channels from plant endomembranes

Plant species	Description of channels	Conductance	Ba^2+^		TEA		Gd^3+^		Ruthenium red		References
			Dose	Effects	Dose	Effects	Dose	Effects	Dose	Effects	
*Marchantia polymorpha*	K^+^ permeable SV channels active at $\left[ {{\text{Ca}}_{\text{cyt}}^{ 2+ } } \right] = 100$ µM	18 pS at 100 mV (100_cyt_/10_vac_ mM K^+^)	3 mM	Channel activity similar as before treatment	3 mM	Blockage	200 µM	Blockage	50 µM	Bursts of flickering type of activity; increase in the open probability (by 67%) and decrease in the conductance (by 14%)	
*Beta vulgaris*	SV channels from tap root active at $\left[ {{\text{Ca}}_{\text{cyt}}^{ 2+ } } \right] = 100$ µM	~130 pS at 100 mV (symmetrical100 mM K^+^)	10 mM	50–70% blockage of the whole vacuole currents	10 mM	20–50% blockage of the whole vacuole currents			0.1–1 µM	Long term closures at voltages lower than ~50 mV and blocks of flickering at higher voltages	Pottosin et al. (1999), Hedrich and Kurkdjian (1988)
									3–5 µM	Blockage	
*Bryonia dioica*	Calcium selective rectifying channels from endoplasmic reticulum. Ca^2+^/K^+^ selectivity ~6.6	29 pS (symmetrical 50 mM Ca^2+^)					10 µM (IC_50_ = 1.1 µM)	Blockage when Gd^3+^ is applied only to the entry (cis) but not exit (trans) side of the channel			Klusener et al. (1995)
*Chenopodium rubrum*	SV channels from hypocotyl cells active at $\left[ {{\text{Ca}}_{\text{cyt}}^{ 2+ } } \right] = 100$ µM	50–70 pS (symmetrical100 mM K^+^)			2.5 mM (IC_50_ = 20 mM)	Fast reduction of the open probability and decrease (by ~ 50%) in unitary conductance					Weiser and Bentrup (1993)
*Eremosphaera viridis*	Outward rectifying channels active at $\left[ {{\text{Ca}}_{\text{cyt}}^{ 2+ } } \right] \ge 100$ nM	150 pS (symmetrical 100 mM K^+^)			10 mM TEA	Reduction of the whole-vacuole currents recorded at +120 mV by 18%					Linz and Köhler (1994)
*Vicia faba*	SV channels from guard cells active at $\left[ {{\text{Ca}}_{\text{cyt}}^{ 2+ } } \right] \ge 100$ μM	280 pS (symmetrical 200 mM K^+^)				TEA can pass through the channels					Schulzlessdorf and Hedrich (1995)

**Table 2:** Effects of anion channel inhibitors (DIDS and Zn^2+^) on the activity of anion permeable channels from plant vacuoles

Plant species	Description of the channels	Conductance	DIDS		Zn^2+^		References
			Dose	Effects	Dose	Effects	
*Marchantia polymorpha*	Permeable to chloride inward rectifying channels active at $\left[ {{\text{Ca}}_{\text{cyt}}^{ 2+ } } \right] \ge 0$ mM	49 pS at –100 mV (104.2_cyt_/14.2_vac_ mM Cl^−^)	50 µM	Blockage	100 µM	Decrease in the open probability recorded a few minutes after application of the inhibitor	
*Conocephalum conicum*	Anion permeable channels activated by 50 mM cytoplasmic Mg^2+^ and low concentration of Ca^2+^ (not added)	32 pS at −100 mV (100_cyt_/104_vac_ mM Cl^−^)	1 mM	Decrease in currents recorded at −100 mV in cytoplasm-out macropatches by ~61%			Trebacz et al. (2007)
*Beta vulgaris*	Time dependent channels passing malate into the vacuole. Mal^2−^/K^+^ selectivity was 6 ÷ 10	≈25 pS at –80 mV (100_cyt_/10_vac_ mM Mal^2−^)			2.5 mM	Blockage of the whole vacuolar currents	Pantoja et al. (1992a)

In conclusion, this study demonstrates for the first time simultaneous activity of potassium- and chloride-permeable channels in the vacuoles of *M. polymorpha*. The first type of channels resemble SV channels known from higher and lower plants, but classification of the other type of channels was more difficult. The activation of chloride-permeable channels was not dependent on the activating factors of vacuolar anion channels known from other plants, also closely related to *Marchantia*, e.g. the liverwort *C. conicum* and the moss *P. patens*. The pharmacological studies of the recorded channels proved high efficiency of the DIDS and Zn^2+^ inhibitors of anion channels in blocking chloride channels and different effects of calcium and potassium channel inhibitors in respect to SV channels.

### *Author contribution statement*

KM conceived and designed research, performed the experiments, analysed the collected data, and wrote the main part of the manuscript. TK reviewed the manuscript, participated in writing, and received a grant from the NCN (National Science Centre) no. 2013/09/B/NZ1/01052. DH reviewed the manuscript. All authors have read and approved the final version of the manuscript.

## Abbreviations

ALMT: Aluminium-activated malate transporter
[Ca^2+^]_cyt/vac_: Concentration of calcium in the cytoplasm/vacuole
E_K/Cl_: Reversal potential for potassium/chloride
SV: Slowly activating vacuolar channel
TEA: Tetraethyl ammonium
TPC1: Two-pore channel 1
